# Multi-model sequential analysis of MRI data for microstructure prediction in heterogeneous tissue

**DOI:** 10.1038/s41598-023-43329-x

**Published:** 2023-10-01

**Authors:** Francisco E. Enríquez-Mier-y-Terán, Aritrick Chatterjee, Tatjana Antic, Aytekin Oto, Gregory Karczmar, Roger Bourne

**Affiliations:** 1 School of Biomedical Engineering, Faculty of Engineering, The University of Sydney, Sydney, 2008 Australia; 2 The Brain and Mind Centre, The University of Sydney, Sydney, 2050 Australia; 3 Department of Radiology, University of Chicago, 5841 South Maryland Avenue, MC 2026, Chicago, 60637 IL USA; 4 Sanford J. Grossman Center of Excellence in Prostate Imaging and Image Guided Therapy, University of Chicago, Chicago, 60637 IL USA; 5 Department of Pathology, University of Chicago, Chicago, 60637 IL USA; 6 Discipline of Medical Imaging Science, Sydney School of Health Sciences, Faculty of Medicine and Health, The University of Sydney, Sydney, 2006 Australia

**Keywords:** Cancer, Magnetic resonance imaging

## Abstract

We propose a general method for combining multiple models to predict tissue microstructure, with an exemplar using in vivo diffusion-relaxation MRI data. The proposed method obviates the need to select a single ’optimum’ structure model for data analysis in heterogeneous tissues where the best model varies according to local environment. We break signal interpretation into a three-stage sequence: (1) application of multiple semi-phenomenological models to predict the physical properties of tissue water pools contributing to the observed signal; (2) from each Stage-1 semi-phenomenological model, application of a tissue microstructure model to predict the relative volumes of tissue structure components that make up each water pool; and (3) aggregation of the predictions of tissue structure, with weightings based on model likelihood and fractional volumes of the water pools from Stage-1. The multiple model approach is expected to reduce prediction variance in tissue regions where a complex model is overparameterised, and bias where a model is underparameterised. The separation of signal characterisation (Stage-1) from biological assignment (Stage-2) enables alternative biological interpretations of the observed physical properties of the system, by application of different tissue structure models. The proposed method is exemplified with human prostate diffusion-relaxation MRI data, but has potential application to a wide range of analyses where a single model may not be optimal throughout the sampled domain.

## Introduction

Modelling of MRI data seeks to characterise measured signals in terms of simplified physical and/or mathematical expressions that can be used to predict structural and functional tissue characteristics. Such models can be broadly categorised as either phenomenological or structural.

Phenomenological approaches focus on mathematical descriptions of the signal, and may or may not have biophysical interpretations. For example, in diffusion-weighted MRI (dMRI), calculation of an apparent diffusion coefficient (ADC) is based on a model that assumes tissue water has a Gaussian displacement probability, and a consequent monoexponential decrease of signal intensity with increasing diffusion weighting (‘b-factor’):

$$\begin{aligned} S/S_0 = e^{-b\cdot ADC} \end{aligned}$$

where *S* and $S_0$ are the diffusion-weighted and unweighted signals respectively. In biological tissue, such water displacement behavior is rare, due to the structural heterogeneity of tissues at both micro and meso scales. In the prostate, the micro scale would roughly encompass cells and subcellular structures, and the meso scale the highly variable glandular and stromal components present within single measurement voxels. The Gleason grading scheme for prostate cancer^1^ is based on ’glandular architecture’—the arrangement and relative volumes of meso scale tissue components.

Phenomenological parameters are often interpreted to reflect tissue structure or function features. The most common example is the assumption that ADC reflects cell density or ’cellularity’. The cellularity interpretation is widespread despite demonstrations that cell type and tissue microstructure, rather than simple cell density, are major determinants of the calculated ADC^2–4^.

The typically non-monoexponential signal attenuation in tissue measurements can be characterised by a kurtosis parameter (*K*):

$$\begin{aligned} S/S_0 = e^{-b\cdot D + b^2\cdot D^2\cdot K/6} \end{aligned}$$

where *D* is a ’kurtosis-adjusted’ diffusion coefficient. *K* can be roughly interpreted to characterise a degree of tissue structure heterogeneity that causes a deviation from a Gaussian displacement probability. An alternative ’stretched exponential’ formalism^5^ is based on an assumed continuum of water pools, each having Gaussian behavior:

$$\begin{aligned} S/S_0 = e^{-(b\cdot D)^\alpha } \end{aligned}$$

where *D* is a ’distributed diffusion coefficient’ and $\alpha$ a ’stretching factor’.

Any water displacement, resulting from either diffusion or incoherent flow, will contribute to dMRI signal attenuation. Flow effects, generally due to blood flow in microvasculature, are most evident in low b-factor measurements. IVIM (Intravoxel Incoherent Motion) analysis^6^ uses a biexponential signal model to separate vascular flow from true diffusion:

$$\begin{aligned} S/S_0 = f\cdot e^{-b (D+D^*)} + (1-f) e^{-b\cdot D} \end{aligned}$$

where *f* is the ’perfusion fraction’ and $D^*$ is a pseudodiffusion coefficient representing flow in randomly oriented capillaries.

Separate from large-displacement flow effects, dMRI measurements that include intermediate and high b-factors also often show a distinct biexponential behavior, indicating the presence of distinct ’slow’ and ’fast’ water diffusion pools. These were initially assumed to represent intra and extracellular water, however several studies have demonstrated this to be an overly simplified interpretation, and biexponential diffusion signal behavior has been reported inside single cells^7,8^. Some biexponential T2 relaxation behavior is thought to reflect distinct pools of ’free’ water and water bound to macromolecules^9^.

Although these models are essentially phenomenological, they may provide some general insight into tissue microstructure features. The IVIM model helps to resolve vasculature from surrounding tissue, and any signal with distinct biexponential decay behavior suggests the presence of two dominant water pools with distinct average water diffusivities, and minimal exchange of water between the pools. Such water pools need not be physically separated in structural compartments. The distinct observed diffusivities simply reflect structural features that determine water displacement probability over the time scale of the dMRI measurement^10^.

A dMRI study of whole prostate specimens ex vivo (where perfusion effects are absent) used a ’stretched biexponential’ formalism to demonstrate that the commonly reported two major water pools each display distinctly non-Gaussian displacement behavior^11^. In the human prostate, signal modelling based on T2 relaxation behavior (’Lumenal Water Imaging’), without diffusion weighting, also indicates the presence of two major water pools, with the signal from the longer T2 pool correlating strongly with histological measurements of gland lumen volume^12^. This correlation does not imply that the long-T2 lumen space is a separate ’compartment’ from the short-T2 environment. The short T2 is likely due to high protein/macromolecule concentration, but the model does not interpret this as either an intra- or extracellular compartment.

In contrast to purely or semi-phenomenological descriptions of MRI signal behavior, microstructural models attempt to define the way the measurement technique (scan protocol) and tissue characteristics interact to produce the measured signal, and to thus predict either specific tissue structural features such as cell size, type, density, shape, and orientation, or the more general features that define pathology. Examples of microstructural models for human prostate MRI include, though are not limited to, VERDICT (Vascular, Extracellular, and Restricted Diffusion for Cytometry in Tumors)^13^ and HM-MRI (Hybrid Multidimensional MRI)^14,15^. These two models will be discussed here because they illustrate some important features and limitations of microstructure modelling. Our exemplar of the multi-model method is based on HM-MRI data.

The different measurement techniques of VERDICT and HM-MRI create constraints on the structural features that the associated models can predict. The VERDICT protocol includes measurements over incremented diffusion times and diffusion weightings, enabling prediction of cell diameter, intra and extracellular volume fractions, and vascular volume. The HM-MRI protocol includes multiple echo times and diffusion weightings to predict the relative volume fractions of epithelium, stroma, and lumen space. An extended version of VERDICT (rVERDICT) adds multiple echo times to increase the ability to resolve tissue compartments based on T2 differences^16^. Here there is some convergence with HM-MRI in terms of scan protocols (data acquisition), while maintaining distinct data modelling. These differences also serve to highlight the important distinction between data acquisition and data analysis choices.

Conceptually, the scan protocol defines and limits the information content of the data. Different data models may extract different parts of the embedded information from the data, and one model may do this better than another for a particular application, however no model can extract absent information. Much of the modelling research literature neglects the possible limitations of scan method, and implicitly assumes that optimisation of imaging applications depends only on determining the best data model for a fixed measurement method^17^.

Both VERDICT and HM-MRI models are simplified descriptors of tissue structure. The model parameters are assigned directly to tissue components despite the known heterogeneity and complexity of the tissue, meaning that multiple tissue features, besides the one assigned, may contribute to the observed model parameter variations.

A wide diversity of microstructure models have been applied to different tissues in recent literature. Their relative performance has been compared in terms of accuracy in prediction of disease (eg. presence and grade of cancer) or histological features, and in more abstract theoretical terms using information criteria. A significant finding of studies that have compared models using information criteria is that there is generally no single ’best’ model, and that the preferred model varies from voxel to voxel throughout the tissue, as demonstrated in brain^18^, prostate^19^, breast^20^, and lymph node tissue^21^. This finding is unsurprising and entirely consistent with the structural heterogeneity of both normal and diseased tissue, yet presents a dilemma for model choice.

The absence of a single best or optimum model, despite the wealth of literature focusing on relative overall performance of specific single models, suggests the need for an approach that can incorporate the predictions of multiple models with weightings related to the appropriateness of each model for the local tissue environment.

Multi-model averaging methods are used extensively in a wide range of fields, from econometrics to ecology and weather forecasting, yet seem rare in medical imaging^22,23^. Brix et al.^24^ describe the application of a model averaging technique to the analysis of simulated dynamic contrast MRI data, although not with the specific aim of addressing tissue heterogeneity.

Applied to tissue microstructure prediction based on MRI data, the multi-model approach would appear limited to models that share some specific parameter (eg. intracellular volume fraction or cellularity). In this case model-averaging could be used to predict a consensus value of the shared parameter (as in Brix et al.^24^).

In this paper we present a method for multi-model analysis, exemplified using in vivo prostate HM-MRI data. By separating the signal description model parameters from specific tissue structure assignment, the method relaxes the conventional direct connection between signal model parameters and tissue structure features - enabling a consensus of diverse signal and structure models, and avoiding the need to select a single ’optimum’ model.

## Methods

**Figure 1:**
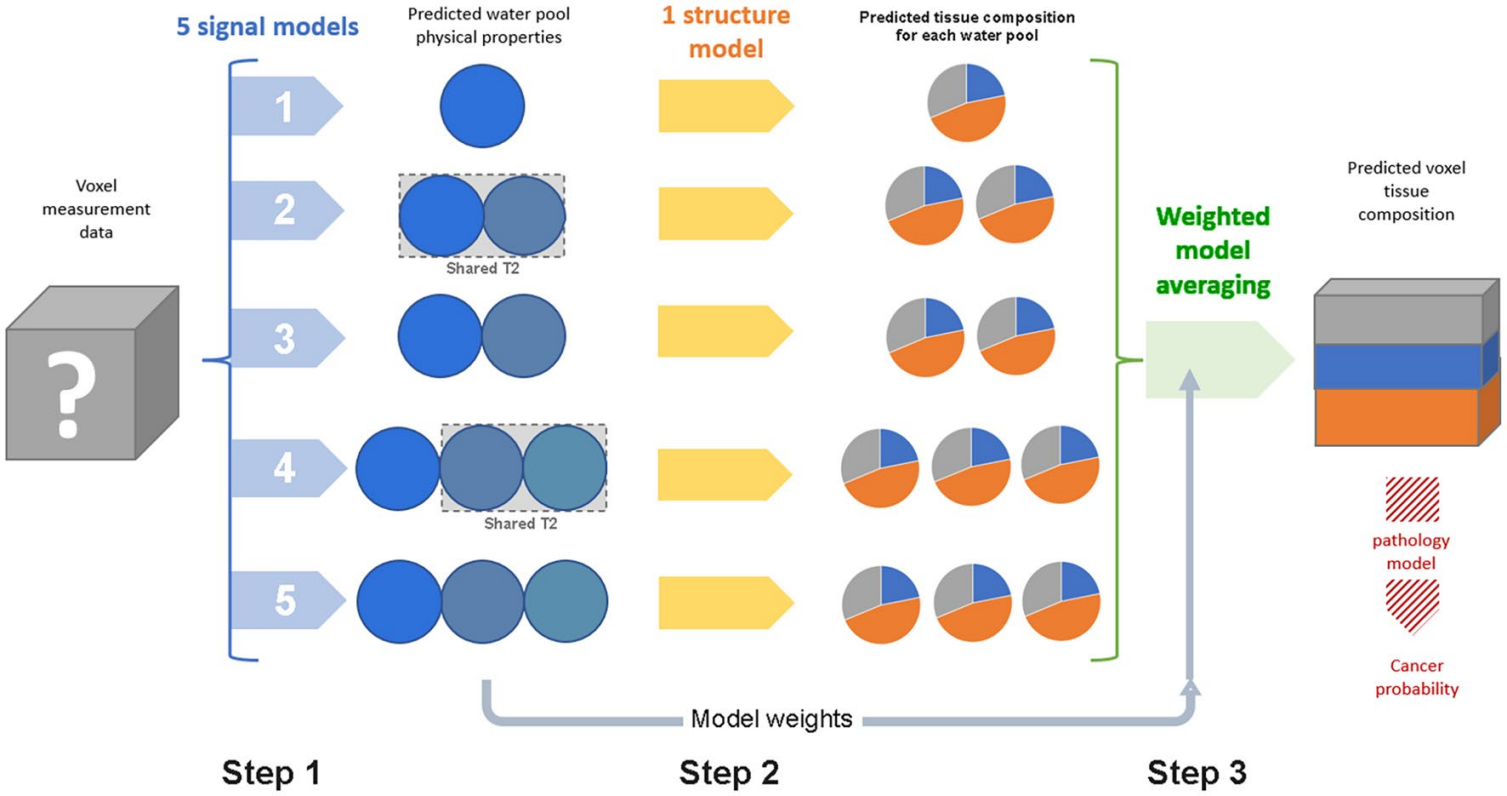
Schematic representation of the sequential modelling approach that separates initial signal analysis from structure assignment. The five phenomenological models are defined in Table 1. In a clinical application, a pathology model may be added to the sequence - for example, to predict the presence of cancer.

The method we illustrate here combines the predictions of multiple models that do not share parameters directly representing tissue properties. We separate the conventional ’direct’ microstructure imaging approach into distinct phenomenological/physical and structural/biological modelling steps (Fig. 1). The generic features of the approach are emphasised in italic.

1. *Multi-model semi-phenomenological signal analysis that predicts the physical properties of the predominant water pools. For each model, Akaike Information Criterion (AIC) is used to estimate model likelihood. Water pools are not attributed to specific microstructural features*. In the example we predict ADC, T2, and fractional volumes ($f_i$) of each water pool in prostate tissue.
2. *Application of a single biological microstructure model to predict the relative volumes* ($f_i$) *of tissue components contributing to the individual water pool physical properties predicted at Step 1*. In the example, we predict epithelium, lumen, and stroma (ESL) volumes, as for HM-MRI. As an alternative example, we predict ’cellularity’.
3. *Microstructure model predictions from Step 2 are combined, with weighting according to model likelihood and water pool fractional volume, to estimate the relative volumes of tissue structural components making up each voxel*.

Using previously acquired in vivo prostate HM-MRI data, we fit five progressively more complex models that describe signals arising from one, two, or three non-mixing water pools with Gaussian diffusion characteristics and a homogeneous T2 value throughout the pool. The simplest, single-pool, model equates to the conventional ADC model with T2 relaxation. Models 2 and 3 each describe two water pools. In Model 2 both pools have the same T2, while distinct T2 values are allowed in Model 3. These ’biexponential’ models are distinct from the IVIM (intravascular incoherent motion) model^6^ in accommodating T2 relaxation, however, in the example data analysed here, the sampled b-values (0, 150, 750, and 1500 s/mm^2^) do not permit accurate assessment of an incoherent perfusion component. Models 4 and 5 describe three water pools with distinct diffusivities. In Model 4 two of these pools share an identical T2 value. Model 5 is equivalent to the mathematical form of the ’standard’ HM-MRI model^14^, but with a broader range of possible diffusivity and T2 values for each water pool (see Supplementary Table S1 online), and no direct assignment of water pools to tissue structure features.

In our exemplar the five models are nested, with the higher order models being less constrained versions of the simpler models. While each of the phenomenological models has a plausible tissue microstructure interpretation (Table 1), this interpretation is not essential, and is not used in this demonstration.

We note that the use of nested models is not a requirement of the method. The only constraint, in our exemplar, is that each model should predict a *T2* and *D* for each water pool.

**Table 1:** Five phenomenological models, with possible biological interpretations. $S_0$ is not fitted as signals are normalised to the intensity in the minimum *b*, minimum *TE* image.

Model	Water pools	Equation	Fitted parameters	Possible tissue composition
1	1	$S/S_0=e^{-bD} \times e^{-TE/T2}$	*D* *T*2	Water in an acellular compartment (e.g. large cystic region)
2	2	$S/S_0=f_1(e^{-bD_1} \times e^{-TE/T2}) + (1-f_1)(e^{-bD_2} \times e^{-TE/T2})$	$D_1$, $D_2$ *T*2 $f_1$	Water in two structurally distinct high-protein environments having identical T2 but different diffusivities (e.g. intra and extracellular water in a high cell density region)
3	2	$S/S_0=f_1(e^{-bD_1} \times e^{-TE/T2_1}) + (1-f_1)(e^{-bD_2} \times e^{-TE/T2_2})$	$D_1$, $D_2$ $T2_1$, $T2_2$ $f_1$	Water in two structurally distinct high-protein environments having different T2 and diffusivities (e.g. intra and extracellular water in a low cell density region)
4	3	$S/S_0=f_1(e^{-bD_1} \times e^{-TE/T2_1}) + f_2(e^{-bD_2} \times e^{-TE/T2_1}) + (1-f_1 - f_2)(e^{-bD_3} \times e^{-TE/T2_2})$	$D_1$, $D_2$, $D_3$ $T2_1$, $T2_2$ $f_1$, $f_2$	Water in three structurally distinct high-protein environments each having different diffusivities but two compartments having identical T2 (e.g. intra and extracellular water in a high cell density region, plus water in lumen space)
5	3	$S/S_0=f_1(e^{-bD_1} \times e^{-TE/T2_1}) + f_2(e^{-bD_2} \times e^{-TE/T2_2}) + (1-f_1 - f_2)(e^{-bD_3} \times e^{-TE/T2_3})$	$D_1$, $D_2$, $D_3$ $T2_1$, $T2_2$, $T2_3$ $f_1$, $f_2$	Water in three structurally distinct high-protein environments each having different diffusivities and T2 (e.g. intra and extracellular water in a low cell density region, plus water in lumen space)

The conventional HM-MRI approach directly and specifically assigns the ESL structural compartments to the three components of the triexponential model. This approach precludes a similar microstructural assignment of the water pools of the mono and biexponential models which may be more appropriate descriptors of the tissue structure in some voxels. To avoid direct assignment, we apply a structural model in the second stage of data analysis to predict the fractional ESL volumes contributing to the predicted water pool *D* and *T*2. The microstructural model assumes that the ESL compartments are internally homogeneous but, throughout the prostate tissue, have a Gaussian distribution of *D* and *T*2 values. The chosen mean and sigma values for the distributions were based on literature reports and, where available, measurements from defined tissue regions validated by histology (see  Supplementary Material online).

By applying the structure modelling to the detected water pools, rather than to the total voxel signal, our method is sensitive to signal variations that would be masked by non-linear multiexponential decay, while incorporation of mono and biexponential models reduces parameter variance in voxels where the higher order models are overparameterised.

More method details are available in  Supplementary Material.

### Image acquisition and preprocessing

In vivo prostate HM-MRI imaging was performed as described previously^15^. Briefly, men with biopsy proven prostate cancer were scanned in a 3T system with cardiac phased-array and endorectal coils. Diffusion weighted images were acquired with all combinations of echo times $TE =$ 57, 70, 150, and 200 ms, and $b =$ 0, 150, 750, and 1500 s/mm^2^, giving a 4 $\times$ 4 array of diffusion-relaxation measurements for each voxel. The image planes were oriented perpendicular to the rectal wall, and approximately transaxial to the prostatic urethra, as guided by sagittal T2 images, to approximately align MRI planes with whole mount histology slices. Diffusion encoding gradients ($\Delta \backslash \delta$ = 21/35 ms) were applied in three orthogonal directions with the resultant images averaged. The raw image data used in the current study were also used in previously described work^25^.

Magnitude mode images were coregistered to reduce motion effects (see Supplementary Fig. S1 online), and Rician noise floor bias was corrected voxelwise according to the method of Gudbjartsson and Patz^26^.

### Step 1. Phenomenological model fitting

Models 1–5 (Table 1) were fitted progressively, starting with the simplest model, with the parameter estimates of the lower order models used to define the parameter limits and starting points for the next higher order model(s) (see Supplementary Fig. S2 online). Non-linear least squares model fitting was performed in Matlab.

For each voxel we calculated the corrected Akaike information criterion^27^:

1 $$\begin{aligned} AICc = N \; \ln \left( \frac{SSE}{N}\right) + 2(p+1)\left( 1+\frac{p+2}{N-p-2}\right) \end{aligned}$$

where *SSE* is the sum of squared residuals, *N* is the number of measurement data points (16 in our example), and *p* is the number of estimated model parameters (2–8 in our example). AICc is used in Step 3 to assign a model weight.

### Step 2. ESL microstructure model fitting

We assume that, across the prostate and the patient population, for any water pool *D* and *T*2 combination the contributions of epithelium, stroma, and lumen (ESL) tissues each have a Gaussian probability distribution with mean and sigma as specified in Table 2. For each water pool *T*2 and *D* value predicted by the phenomenological models, we calculate a Z-score ($Z_E$, $Z_S$, $Z_L$; range 0–1) of the Gaussian distribution for the E, S, and L components. The predicted fractional volume (*x*) of the tissue component contributing to the water pool is then calculated as:

2 $$\begin{aligned} x = \frac{Z_X}{Z_E + Z_S + Z_L} \end{aligned}$$

This step predicts the tissue structure (fractional volumes of the ESL compartments) giving rise to the physical properties of each water pool (Fig. 1, ’Predicted tissue composition for each water pool’).

### Step 3. Weighted model averaging

The AICc values from the five models (Step 1) were used to calculate model weights ($W_m$ = normalised likelihood) according to^28^ (p. 447):

3 $$\begin{aligned} W_m = \frac{e^{-\frac{\Delta _m}{2}}}{\sum _{m=1}^{5}e^{-\frac{\Delta _m}{2}} } \end{aligned}$$

where $\Delta _m$ is the AICc difference between model *m* and the model with lowest AICc value ($AICc_m - AICc_{min}$).

The weighted multi-model ESL prediction for voxel structure was calculated based on the fractional volume of ESL components in each water pool and the associated model weight:

4 $$\begin{aligned} X = \frac{\sum _{m=1}^{5}\sum _{p=1}^{n}W_m \cdot f_m^p \cdot x_m^p}{\sum _{m=1}^5 W_m} \end{aligned}$$

where $X \in \{E,S,L\}$ is the model-averaged fractional volume of a given ESL component, *m* = model number (1, 2, ..., 5), *n* = number of water pools in model *m*, $f_m^p$ = fractional volume of water pool *p* in model *m*, and $x_m^p$ = fractional volume of ESL component *x* in pool *p* of model *m*.

**Table 2:** Assumed Gaussian probability distributions for contributions of the three compartments of the ESL microstructure model (details in  Supplementary Material online).

	E	S	L
D $(\upmu \textrm{m}^2/\textrm{ms})$ Mean (sigma)	0.3 (0.2)	1.3 (0.5)	2.5 (0.5)
T2 (ms) Mean (sigma)	60 (30)	220 (90)	550 (150)

### Cancer probability prediction

As a demonstration of the possible application of a pathology model to the predicted ESL composition of the prostate tissue, we calculated a hypothetical cancer probability ($P_{Ca}$) for each voxel based on the clinical observation that prostatic adenocarcinoma is characterised by proliferation of epithelial cells and occlusion of lumen space^29^:

5 $$\begin{aligned} P_{Ca} = E\cdot (1-L) \end{aligned}$$

where *E* and *L* are the predicted fractional volumes of epithelium and lumen. Stroma volume is omitted as it correlates poorly with presence and grade of prostate cancer^2^, and is redundant given the epithelium and lumen fractional volumes ($E+S+L = 1$). This model is a simple extension of the binary cancer prediction model of conventional HM-MRI^14^.

### Alternative ‘cellularity’ model

To illustrate the ease of implementing an alternative microstructure model, we replaced the ESL model with a hypothetical simple cell density model. This model assumes intra and extracellular water having a Gaussian distribution of *T*2 and *D* values with mean and sigma as shown in Table 3. In this example ’cellularity’ is presented as intracellular volume fraction, and has a range of 0–1.

**Table 3:** Assumed Gaussian probability distributions for *D* and *T*2 in the compartments of the ’cellularity’ microstructure model.

	Intracellular water	Extracellular water
D $(\upmu \textrm{m}^2 /\textrm{ms})$ Mean (sigma)	0.5 (0.3)	1.2 (0.3)
T2 (ms) Mean (sigma)	50 (20)	150 (40)

Note that, as this cellularity model is presented solely as an example of the ability of the general method to employ alternative structural models, the *D* and *T*2 values are somewhat arbitrary and not based on literature or measurement data.

### Histology data

For the exemplar data presented, there was no precise alignment of histology sections with MRI planes. Radical prostatectomy specimens were sliced in 3 mm thick sections transverse to the posterior surface of the prostate. Thus the histology images lie approximately parallel to the the MRI data, although not necessarily co-planar. We note that rigorous histological validation of MRI studies is an ongoing challenge^30^, beyond the scope of the general method proposal presented here. In the figures presented here, we show the histology image which has the best overall anatomical match to the MRI features.

## Results

**Figure 2:**
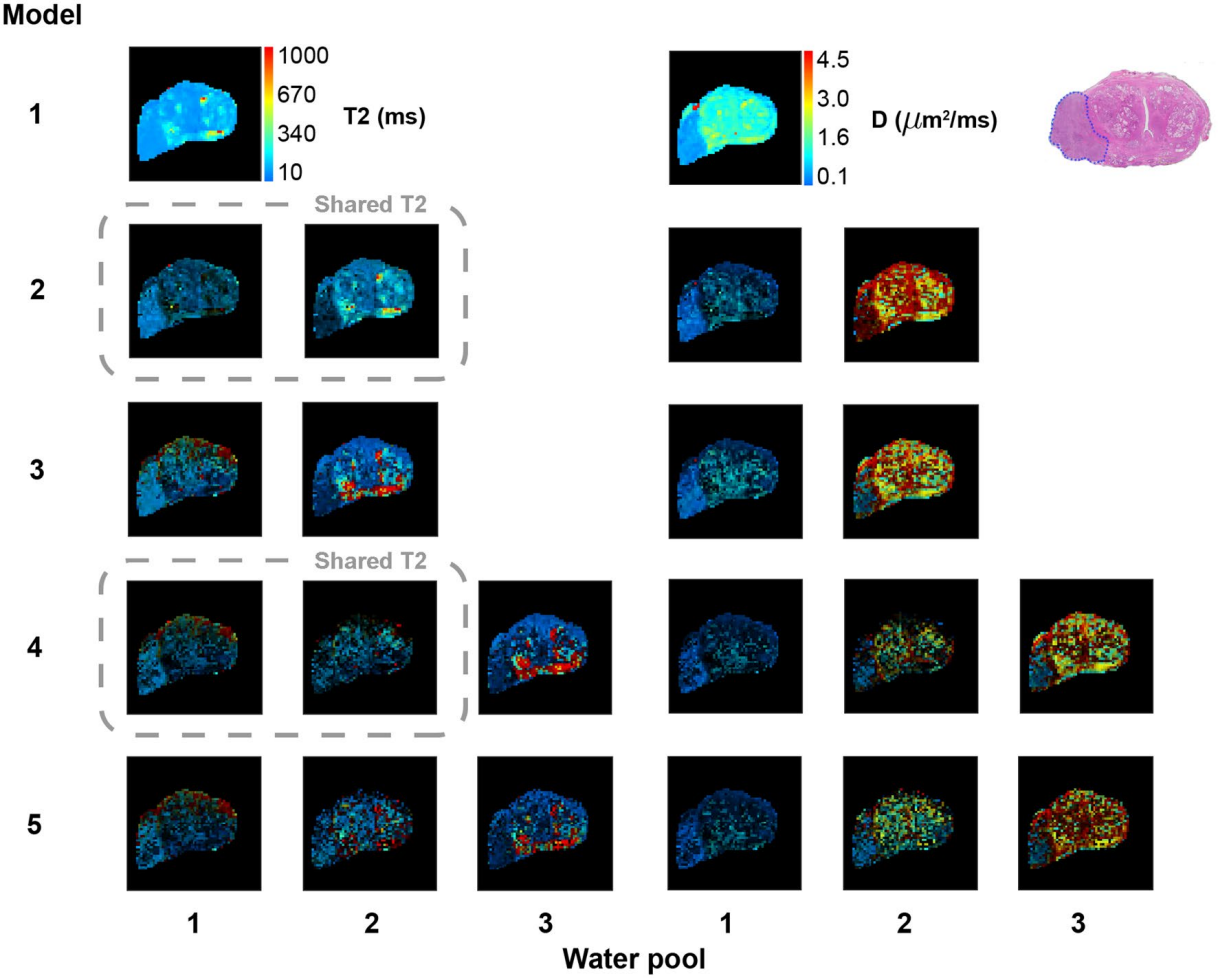
Voxelwise water pool *T*2 and *D* predictions from five phenomenological models. Pixel colour indicates water pool *T*2 or *D* value, while brightness is scaled according to fractional volume of the water pool. Dashed grey boxes connect models that share a single *T*2 parameter across two water pools (note that the fractional volume pixel brightness weighting means that these *T*2 maps will not appear identical, despite the shared T2 value). The histology section lies in approximately the same plane as the MRI slice, and shows a large high grade tumour in the right peripheral zone (left side of image). Unweighted *D* and *T*2 maps are provided together with fractional volume maps in  Supplementary Material (see Supplementary Fig. S3 online).

Figure 2 shows predicted water pool *T*2 and *D* values from the five phenomenological models across a transverse slice of a human prostate that includes a large high grade tumour. The pixel colour represents *T*2 and *D* value, while the pixel brightness is weighted according to water pool fractional volume. This weighting emphasises the parameter values of voxels where signal to noise ratio (SNR) for the associated water pool is high and the effect of noise on parameter variance is low. When the fractional volume of a water pool is low the associated SNR will be low, leading to unreliable estimates of *T*2 and *D*. These voxels are darkened in the maps. The corresponding unweighted *D* and *T*2 maps are provided together with fractional volume maps in the  Supplementary Material.

**Figure 3:**
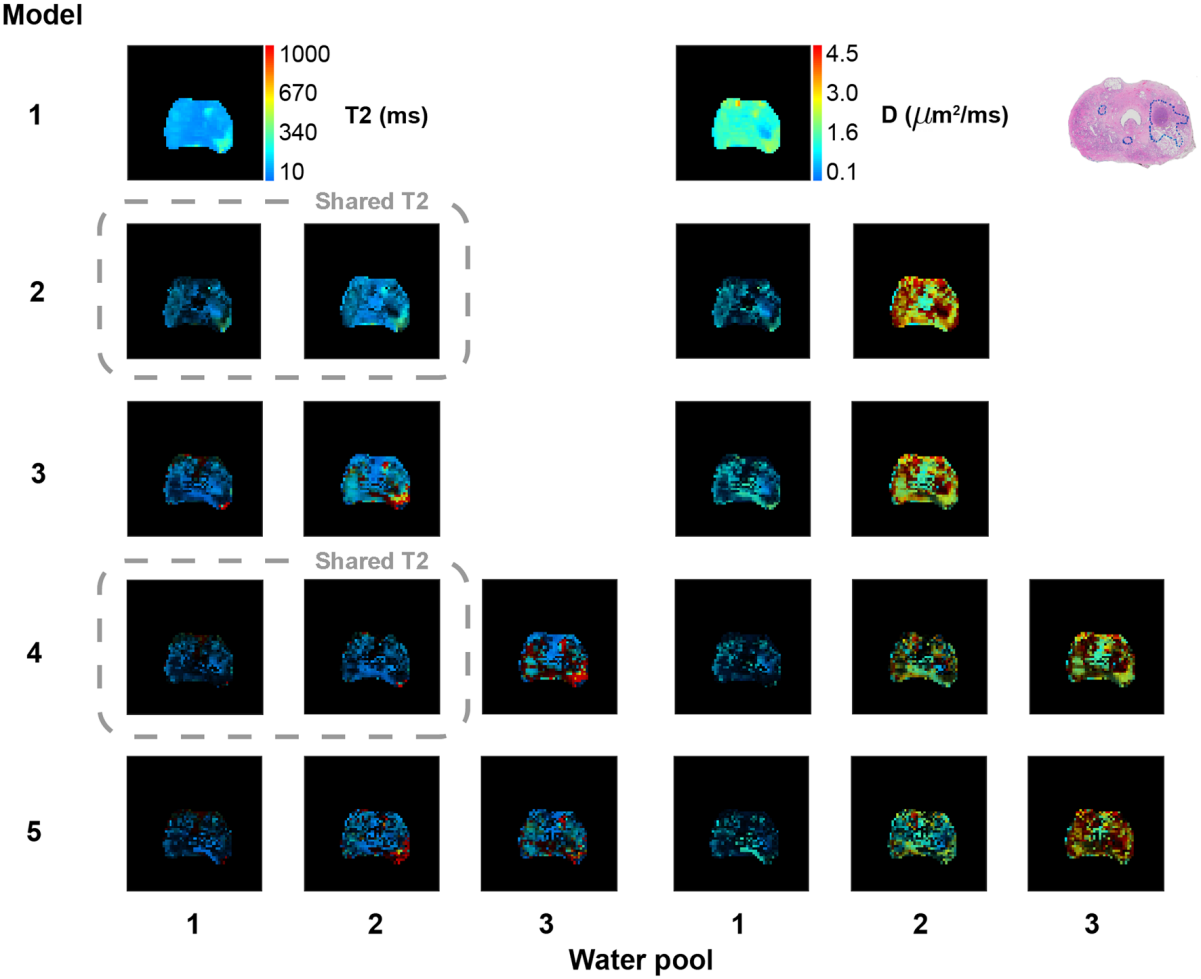
Patient 2 voxelwise water pool *T*2 and *D* predictions from five phenomenological models. Details as for Fig. 2.

**Figure 4:**
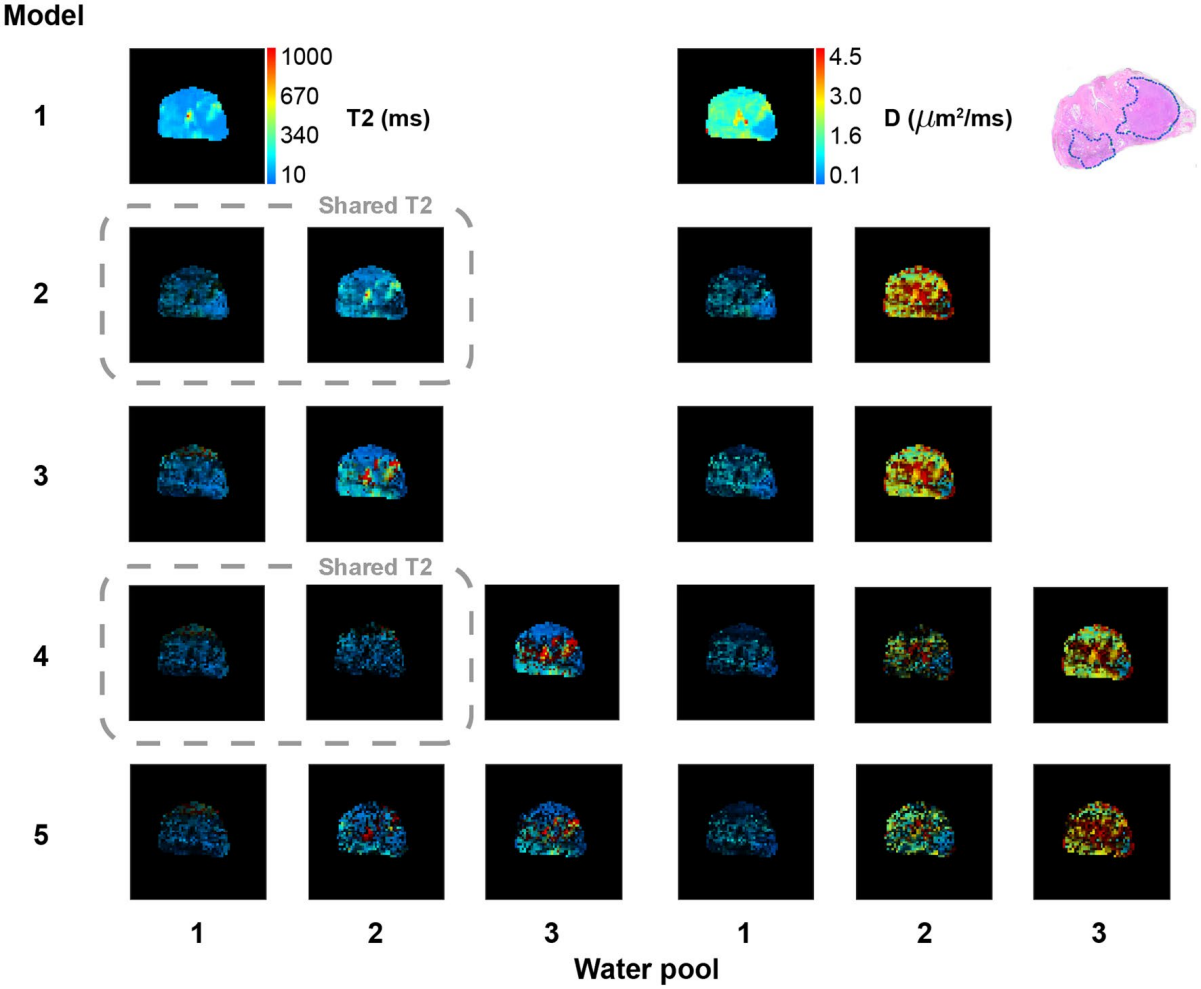
Patient 3 voxelwise water pool *T*2 and *D* predictions from five phenomenological models. Details as for Fig. 2.

Figures 3 and 4 provide similar data for two further patients.

The *T*2 maps indicate that for all models the large majority of voxels show water pool *T*2 estimates that are biopyhsically plausible and lie inside the allowed model fitting range of 10–1000 ms. The two-pool models show distinct differences in *T*2 values between the two pools. In comparison, the three-pool models show relatively minor differences between pools 1 and 2, with pool 3 having similar *T*2 to pool 2 of the two-pool models.

The two-pool models show distinct diffusivity differences between the pools. Diffusivity values are mostly biophysically plausible, although in Models 2 and 3 there are significant areas with D > 3.1 $\upmu \hbox {m}^{2}$/ms (the free diffusion maximum at body temperature). These high *D* regions, which, based on prostate anatomy, are unlikely to represent true perfusion effects, are much reduced in Models 4 and 5.

All models show clear differences between the large high grade (Gleason $4+5$) tumour in the right peripheral zone (PZ) and the non-cancerous regions of the prostate. However, the two- and three-pool model parameter values show much more variation within the benign tissue than the single pool ‘ADC’ model. The generally high *T*2 values in the PZ are consistent with conventional T2-weighted imaging, where benign PZ tissue is typically hyperintense relative to the central prostate. As there was no precise alignment of histology sections and MRI planes^30^, a close match between the location and boundaries of the tumour in the histology and MR images is not expected.

**Figure 5:**
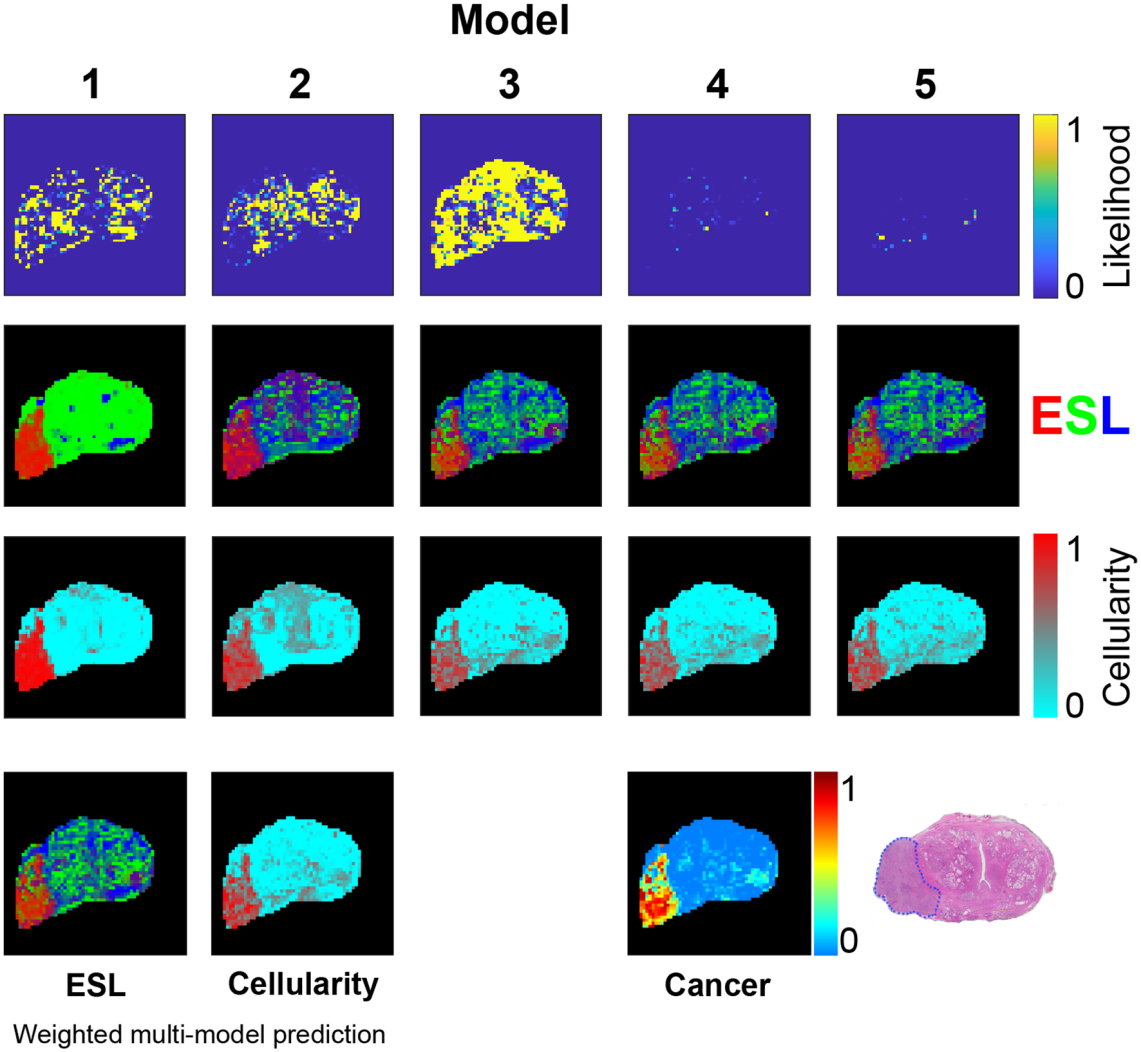
Model likelihoods based on AICc and predictions of ESL fractional volumes from each model. RGB values for each ESL voxel are proportional to fractional volumes of epithelium (red), stroma (green), and lumen (blue). Predictions of the five models are combined, with weighting according to model likelihood, to produce the weighted multi-model ESL prediction. ‘Cellularity’ maps are based on application of a two-component intra- extracellular water model as an alternative to the ESL model. ESL cancer probability is based on a pathology model (Eq. 5) applied to the ESL weighted multi-model prediction. The histology section lies in approximately the same plane as the MRI data and shows a large high grade tumour (outlined in blue) in the right PZ.

Figure 5 shows maps of model likelihood, and corresponding predictions of the partial volumes of epithelium, stroma, and lumen space predicted by application of the ESL microstructural model to each of the phenomenological model water pool properties. For the multi-pool models, the ESL predictions from each pool in the model are averaged, with weighting according to pool fractional volume. In addition, the model-averaged ESL map is provided with weighting based on individual model likelihoods. The single-model ESL maps illustrate how selection of a single model would influence the structure prediction, and the degree to which the prediction from weighted model averaging differs from the single model predictions.

To demonstrate the ability to apply alternative structural models to the multi-model water pool predictions, results from the alternative ’cellularity’ structural model are shown in the third row of Fig. 5. In this model a cellularity of 1 indicates that all water is intracellular, and a cellularity of 0 indicates that all water is extracellular (zero cell density).

The likelihood maps indicate that Model 3 provides the best signal description in a little more than half the voxels, with the majority of remaining voxels best described by the simpler two-pool Model 2 or the single pool Model 1. The three-pool models have very low likelihoods in all but a few voxels—a result that is unsurprising given the small number of measurements relative to the number of model parameters, and the relatively low SNR of the in vivo measurements.

Despite their low likelihoods, the three-pool models predict very similar ESL values to the two-pool models—suggesting that the multi-step structure modelling approach may compensate overparameterisation.

The weighted multi-model ESL map shows small differences from each of the individual model ESL maps, and patterns that are consistent with the approximately aligned histology section. Notably, highly cystic (acellular) regions of the PZ and central zone roughly correspond to high lumen volume regions (dark blue) in the ESL maps. Model 1 predicts very high stromal density throughout the non-tumourous prostate—a result not supported by the histology section. In contrast, the two and three pool models, and the weighted combination, predict a heterogeneous mixture of lumen and stroma, in good agreement with the histology and normal prostate anatomy.

Figure 5 also presents a possible pathological interpretation of the multi-model ESL prediction by calculating a hypothetical cancer probability based on fractional volumes of epithelium and lumen space (Eq. 5). Notwithstanding the imperfect alignment of histology section with MRI slice, the probability map shows a qualitatively good agreement with the histologically defined cancer extent.

The cellularity model correctly predicts high cell density in the tumour, but in contrast to the ESL model, fails to reflect normal anatomical heterogeneity in the peripheral and central regions of the prostate.

**Figure 6:**
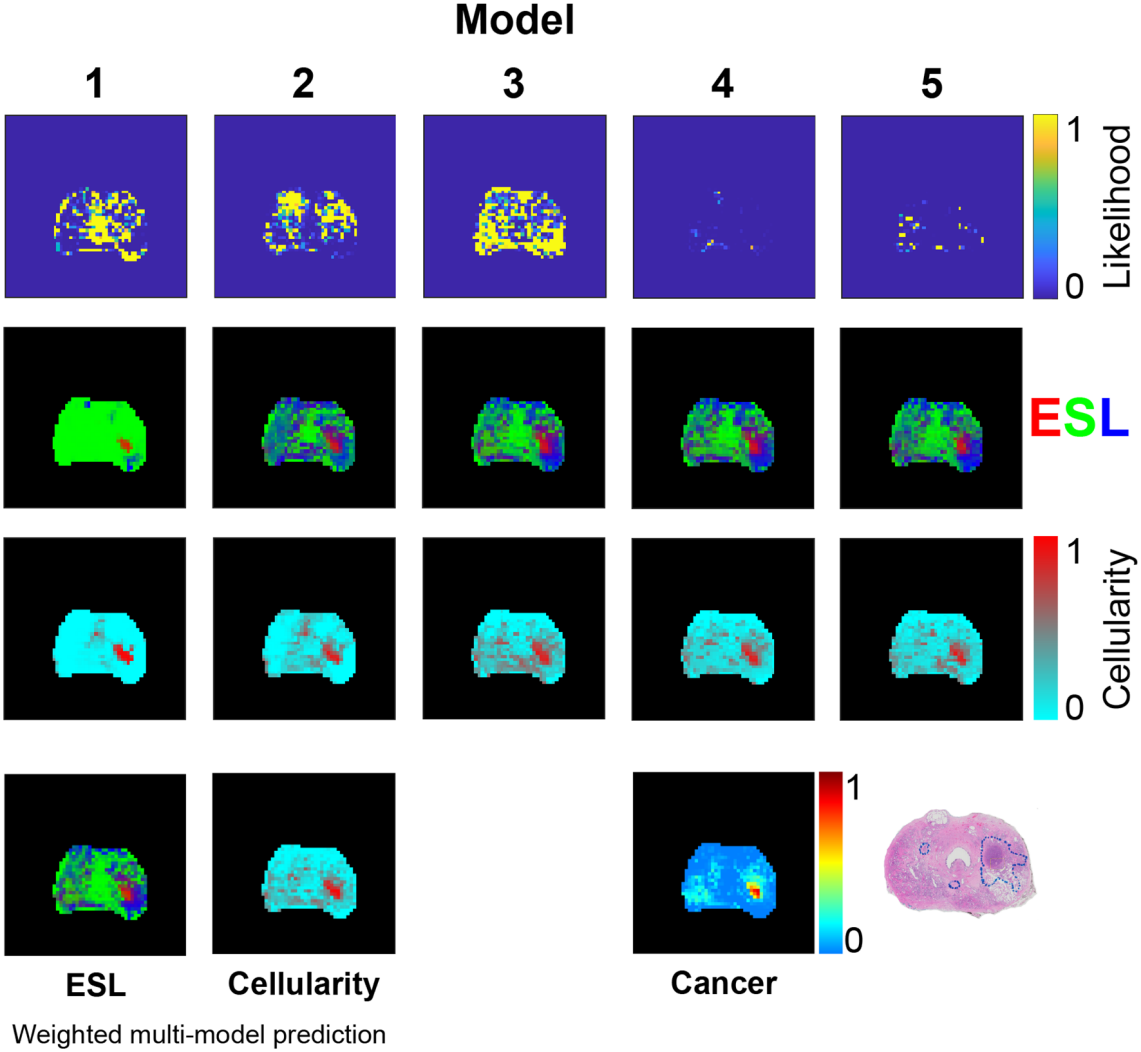
Patient 2 model likelihoods based on AICc and predictions of ESL fractional volumes from each model. Details as for Fig. 5.

**Figure 7:**
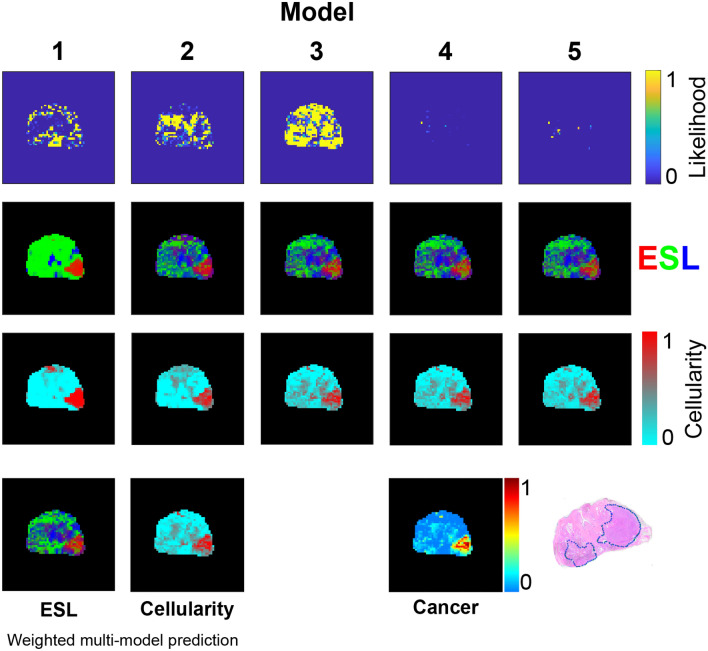
Patient 3 model likelihoods based on AICc and predictions of ESL fractional volumes from each model. Details as for Fig. 5.

Results from the two other patients are provided in Figs. 6 and 7 . The broadly consistent and anatomically plausible ESL predictions across the three patients suggest that the assumptions of *T*2 and *D* statistical distributions used for the structure model (Table 2) are reasonable.

**Figure 8:**
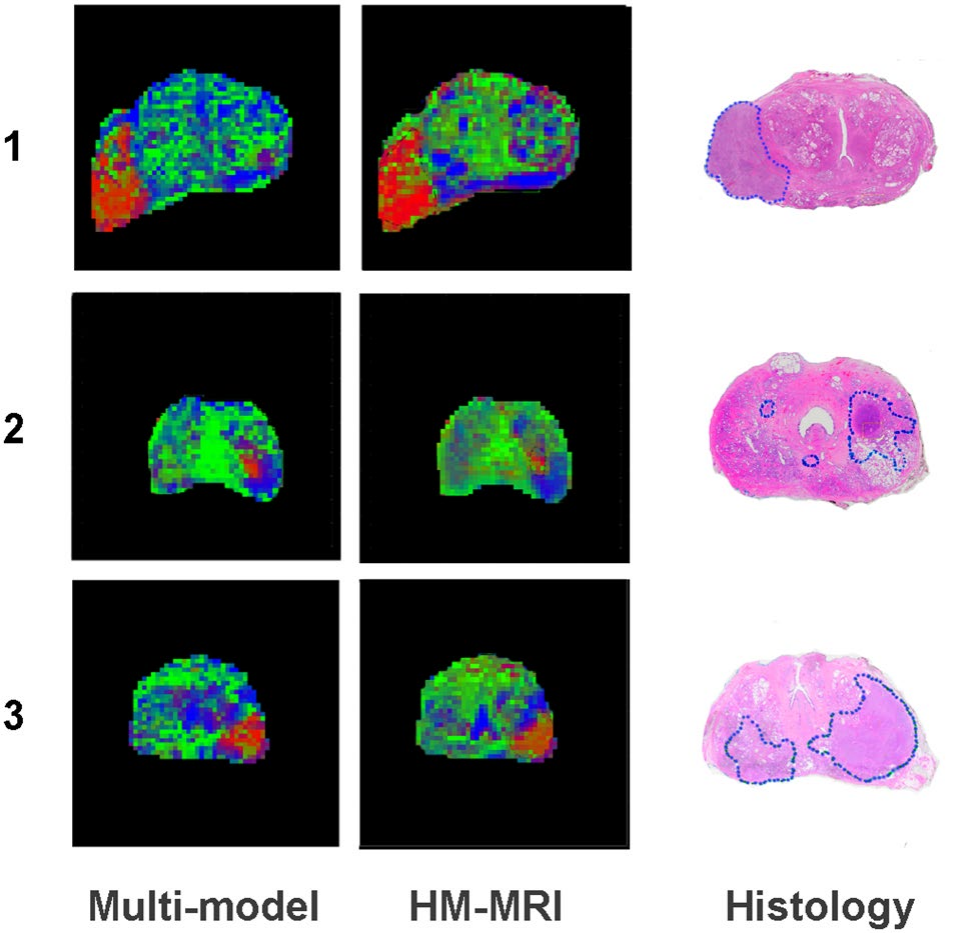
Qualitative comparison of ESL gland component predictions from the multi-model method and the three-component HM-MRI method^15^. Data are shown for three patients with approximately aligned histology sections (regions of cancer are outlined in blue). Note that, due to the imperfect alignment of histology and MRI sections, close correspondence of MRI-predicted structure and histology is not expected.

Figure 8 provides a qualitative comparison of ESL structure predictions from the multi-model method and the ’conventional’ three-compartment HM-MRI model^15^ in three patients. As the alignment of histology sections and in vivo MRI slice planes is imperfect, close correspondence of MRI-predicted structure and histology is not expected. However, based on general prostate anatomical features, there appear to be some advantages of the multi-model approach. In Prostate 1 (i.e., from patient 1), the HM-MRI method appears to overestimate the fractional volume of epithelium (bright red) in the tumour, and the fractional volume of lumen space (dark blue) in the posterior peripheral zone. HM-MRI also, implausibly, suggests high epithelium volume at the left and anterior edges of the prostate. In Prostates 2 and 3 the multi-model prediction appears to show the dense part of the left side tumour more distinctly than the HM-MRI prediction.

## Discussion

We have proposed a method for predicting tissue microstructure from MRI data based on combining the predictions of multiple models, with weighting according to calculated model likelihood. Conceptually, our method avoids direct assignment of tissue structure features to signal characteristics. The multiple phenomenological models account for both meso and micro scale heterogeneity of the signal source (usually one voxel) in terms of pools of water that may, or may not, have distinct physical properties. We then apply a single tissue structure model to predict the microstructural features that give rise to the signal behavior of each detected water pool, and aggregate these pool predictions to summarise the structure of the signal source (voxel).

Our approach differs significantly from Brix et al’s multi-model analysis of simulated dynamic contrast enhanced MRI data^24^, in that we do not directly assign tissue properties to the model parameters, and that we apply the likelihood-based model weightings, not to the signal models, but to the tissue structure predictions made subsequent to the initial semi-phenomenological signal modelling. Below we discuss some of the underlying assumptions and potential refinements and modifications of our approach.

Choice of the five phenomenological models was based on what we considered likely biophysical properties of water pools in prostate tissue. Models with constrained/shared diffusivity and free *T*2 were not tested as we assumed that while many equally protein-dense tissue structures may exist, giving similar *T*2 but varying *D*, the alternative of equal diffusion restriction/hindrance but varying protein density is unlikely in animal tissue. Either way, we emphasise that these five models are presented simply to illustrate the general multi-model approach, and the models are not specifically nor exclusively appropriate to the prostate tissue from which the exemplar data were acquired.

**Figure 9:**
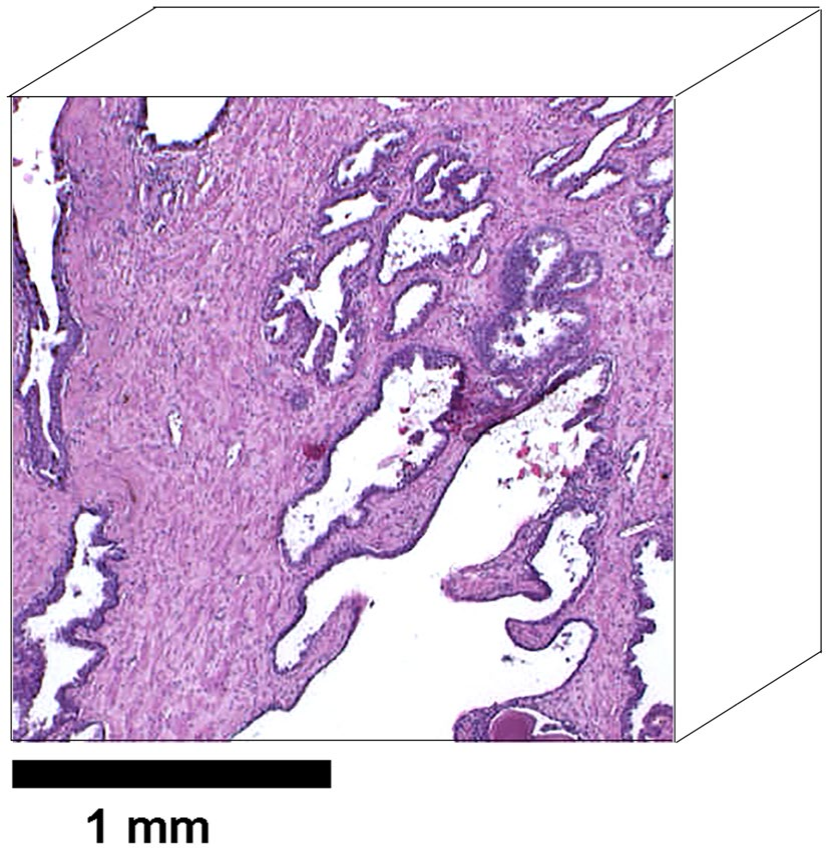
Prostate histology section illustrating possible meso scale tissue heterogeneity giving rise to distinct water pools in a voxel of size 2$\times$2$\times$2 mm. In this example there are plausibly three distinct environments: acellular cystic space; dense non-glandular stroma; and loosely packed glands.

Our phenomenological ’water pools’ approach accounts for mesoscopic tissue heterogeneity. Numerous experimental works have demonstrated that multiple signal components are often simplistically overinterpreted as representing distinct microstructural compartments. However, these studies are mostly based on mesoscopically homogeneous tissues and cell suspensions. Many tissues are highly heterogeneous at the meso scale, such that in any single voxel there may be large regions of very different structure. Fig. 9 illustrates such meso scale structure heterogeneity in a 2 $\times$ 2 mm section of prostate tissue that could represent a cross-section of a typical MRI voxel. This section, as a 3-D volume, could plausibly give rise to three distinct water pool signals: (1) free water in the large cystic spaces; (2) highly hindered and restricted water in the dense stroma; and (3) water in the complex of small glands. At the microscopic scale, both the stroma and glandular spaces would be internally heterogeneous, and very different from each other. A three-component signal model might directly and erroneously interpret these three water pools as ‘intracellular’, ‘interstitial’, and ‘lumenal’, on the basis of distinct average diffusivities, when the first two pools actually contain both intracellular and interstitial water, and the second water pool contains lumen spaces. This example illustrates how, depending on mesoscopic tissue structure and voxel size, distinct water pools (signals) may be observed and yet be inaccurately characterised by an applied microstructure model.

While we did not explicitly model a vascular signal component, several of the models predicted a significant number of voxels with high *D* values (D > 3.1 $\upmu$m^2^/ms) in one water pool. The high *D* values are generally less concerning when assessed in conjunction with the corresponding model likelihood maps. In Fig. 2 the very high *D* values seen in the anterior of the prostate in pool 2 of Model 2 mostly occur when Model 2 has a low likelihood. The high *D* values are much less frequent in pool 2 of Model 3 where the likelihood is high. The low likelihood of the Model 2 predictions in this region result in a low weighting in the multi-model structure prediction. In the combined ESL model prediction of Fig. 5 the predicted structure in this region is a mixture of stroma and lumen—generally consistent with the structures present in the corresponding histology sections. Nevertheless, there is possibly an excess of high *D* voxels, and it is unlikely that most prostates would have such a high vascular volume. Similar ‘excess vasculature’ results have been reported for VERDICT, which specifically aims to quantify the vascular component. This is an issue that requires deeper investigation, although we believe it is outside the scope of this paper.

Our ESL microstructure model assumes a Gaussian probability distribution for the contributions of individual E, S, and L components to an observed or predicted combination of water pool *D* and *T*2 values. Where available, we based our choice of probability distributions on related literature data, as detailed in Supporting Material. Other probability distributions may be more accurate, and could potentially be ascertained in a specific study that employs precision alignment of MRI and histology data. Nevertheless, the distributions defined in Table 2 produced ESL microstructure maps in generally good agreement with conventional HM-MRI results from the same data^15^. Given the uncertainties in histological validation of studies based on in vivo imaging^30^, we cannot at this stage provide a quantitative comparison of the structure prediction accuracy of the multi-model ESL method versus conventional HM-MRI.

As for the five signal models, the ESL and ’cellularity’ models are presented here only as exemplars of the flexibility of structure model assignments that is made possible when signal model parameters are not directly assigned to tissue features. While we focus on the ESL microstructure model, the proposed separation of signal modelling from tissue microstructure modelling opens the door to application of alternative biological interpretations of the signal model predictions. The two-component ’cellularity’ model was provided as an example of an alternative to the ESL model. The ESL model does not account for any perfusion effects from vascular water (cf. VERDICT), but this could be added as a fourth, high apparent diffusivity component with mean *T*2 = $\sim$350 ms, as expected for blood^9^.

Where the signal acquisition protocol permits, higher order microstructure models could be applied. An obvious candidate is the addition of a third ’time-dependence’ or restriction radius coordinate to the *D* and *T*2 distributions to enable characterisation of diffusion-restricting structure features^10,13^, as used in VERDICT.

We used AICc as the information criterion for calculation of model weights. This choice is somewhat arbitrary, although model ranking based on AICc has previously been shown to correlate well with an independent test of model prediction error in a dMRI study of whole prostates ex vivo^31^. The ’correction’ to standard AIC aims to account for situations (such as our higher order models) where number of samples (*TE* and *b* values) is low relative to number of model parameters. Other corrections (penalty terms), or information criteria such as BIC (Bayesian information criterion), might be considered. BIC tends to favour simpler models than AIC. However, discussions of best information criteria are generally focused on model selection and its implications^32^, rather than weighting for multi-model prediction.

This paper focuses on a proposed generic method, and provides exemplars using existing MRI data. Validation of the sequential multi-model method in terms of a quantitative assessment of prediction accuracy will be highly application-specific, and is beyond the scope of the presented work. Rigorous histological validation, while particularly challenging^30^, is important future work.

In summary, we have proposed a method for sequential multi-model analysis of complex MRI data where no single model accounts for both meso and micro scale tissue structure heterogeneity. In addition to implementing a method for prediction from multiple models, we suggest the proposed sequential separation of phenomenological signal modelling from structure assignment or clinical interpretation has potentially broad application in biology and other fields.

### Supplementary Information

Supplementary Information.

## Data Availability

Data are available on application to the corresponding author.
