# Supplementary material for: Multi-model sequential analysis of MRI data for microstructure prediction in heterogeneous tissue
Source: Sci Rep. 2023 Oct 1;13:16486. doi: 10.1038/s41598-023-43329-x (PMC10543593; doi:10.1038/s41598-023-43329-x)
Supplement: Supplementary file 1 — Supplementary Information. [file 41598_2023_43329_MOESM1_ESM.docx]

**Methods**

**Image Acquisition**

Participants underwent preoperative 3T multiparametric MRI (Ingenia; Philips Healthcare) using a combination of a six-channel cardiac phased-array coil placed around the pelvis and an endorectal coil (Medrad; Bayer Healthcare). The HM MRI sequence consisted of a spin-echo module with diffusion sensitising gradients (δ/Δ = 21/35 ms) placed symmetrically about the 180° pulse, followed by single-shot echo-planar imaging readout. Axial HM MRI images were acquired with all combinations of echo times of 57, 70, 150, and 200 ms and b values of 0, 150, 750, and 1500 s/mm^2^, which resulted in a 4 x 4 array of data associated with each voxel. Axial HM MRI images were oriented perpendicular to the rectal wall, as guided by sagittal images to align MRI scans more closely with wholemount histologic slices. Diffusion encoding gradients were applied in three orthogonal directions with the resultant images averaged. Fat saturation was performed using spectral adiabatic inversion recovery. The MRI parameters were as follows: in-plane resolution, 1.5 x 1.5 mm; imaging matrix, 120 x 120; field of view, 180 x 180 mm; repetition time, 5 s; number of slices, 18; slice thickness, 3 mm; reconstruction matrix, 128 x 128. The acquisition time was 10–12 minutes. Further details are provided in Chatterjee et al (2022).

**Image registration**

Prior to registration images were cropped to just include the largest cross section of the prostate plus approximately 5 mm of left/right and anterior/posterior tissue. Data precorrection was applied to the MR images prior the model fitting in a two-step process using MATLAB (version R2022a). Images were firstly coregistered using an intensity-based image registration method with affine transformations. The optimizer initial radius was set to 6.3 $\times$10^-4^ and the maximum number of iterations to 3$\times$10^5^. For the registration, the MR image sequence with lowest b and TE value served as the reference image (Fig. S1). From this, and to reduce misalignment errors due to patient movement, all other images with b = 0 and different TE sequences were firstly coregistered such that sequence TE = TE1 was used to align sequence TE = TE2, with TE2 > TE1. Once all b = 0, TE sequences were aligned, these images serve as the reference image for all the other sequences with same TE but different b values. This stepwise process ensures that each imaged is registered to the next most similar image in terms of T2 and diffusion attenuation.

| 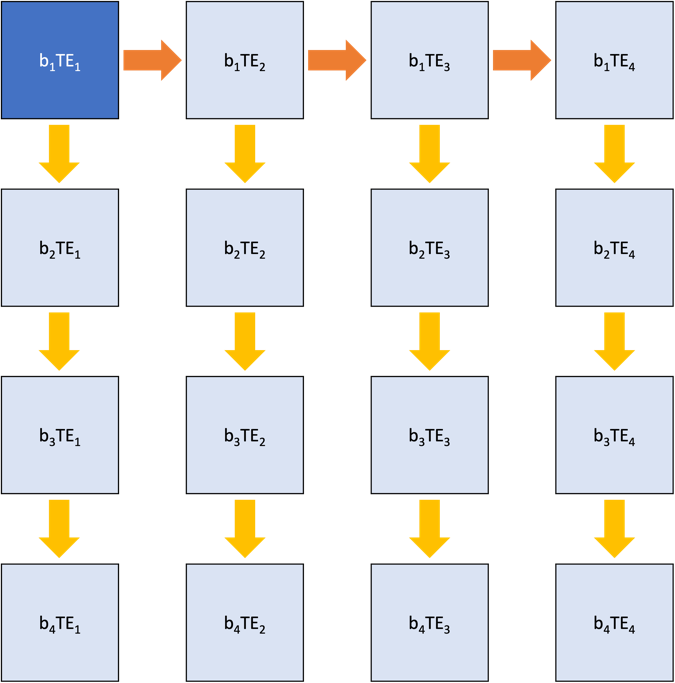 |
| --- |
| **Fig. S1. Image registration sequence**  Image registration was performed using the image sequence with lowest TE and b value ($b = 0$ s/mm^2^) as the reference image (dark blue). The sequence followed during registration is indicated by the orange and yellow arrows: 1) Firstly, all images with same (lowest) b value were coregistered using the image on the left as the reference image (orange arrows); 2) Once all these sequences were registered, each image on the top row was used as the reference image for its corresponding column. For a given image sequence, the image above was used as the reference image (yellow arrows) |

**Noise floor correction**

Prior to model fitting, signal data from each voxel was adjusted to compensate Rician noise bias according to the method of Gudbjartsson (1995). The noise level was taken as the standard deviation of signal intensity in an ROI drawn in air inside the endorectal coil adjacent to the prostate.

**Phenomenological model fitting**

After image registration and noise bias reduction, a mask was manually defined for each prostate slice to include only prostatic tissue. Model fitting was performed only on intraprostatic voxels as defined by the mask.

Five nested models were fitted progressively to the data (Fig. S2). Starting from the simplest model (Model 1), one D parameter and one T2 parameter are estimated. The fitted D value of Model 1 is used to define the lower and upper D limits for the subsequent two models (Models 2 & 3) and the D estimates of Model 2 to define the initial D points for Model 3. A similar approach is then followed for Models 4 and 5, where the D limits (lower and upper) for both models are defined by the D estimates of Model 3 and the initial D values for Model 5 are obtained from Model 4. For T2, limits were always set to 10-1000 ms across models and compartments. Constraining the T2 limits did not produce significant differences on the D and T2 maps. For models sharing the same number of water pools, the initial estimates were given by the previous model but otherwise the initial estimates were the midpoint of the full range.

Model fitting was performed in MATLAB (version R2022a) using the ‘fit’ function and a non-linear least squares method. The ‘Trust-Region’ algorithm was used during fitting and the ‘bisquare’ robust method employed to limit the effect of outliers. The maximum change difference was set to 0.001 and the maximum number of function evaluations and iterations were set to 60,000 and 40,000, respectively. All fittings were performed using MATLAB parallel-computing tools, with 6 CPU cores running on an Intel Xeon E5-2680 V3 @ 2.50 GHz, and with a typical execution time of 30-40 min, depending on number of voxels in the prostate slice.

**Supplementary Table S1. Differences from standard HM-MRI model**

| **Parameter** | **Model 5 allowed range^*^** | **HM-MRI allowed range** |
| --- | --- | --- |
| D_1 (HMMRI ‘epithelium) | 0.1 – 4.5 µm^2^/ms | 0.3 – 0.7 µm^2^/ms |
| D_2 (HMMRI ‘stroma’) | 0.1 – 4.5 | 0.7 – 1.7 |
| D_3 (HMMRI ‘lumen) | 0.1 – 4.5 | 2.7 – 3.0 |
| T2_1 (HMMRI ‘epithelium) | 10 – 1000 ms | 20 – 70 ms |
| T2_2 (HMMRI ‘stroma’) | 10 - 1000 | 40 - 100 |
| T2_3 (HMMRI ‘lumen) | 10 - 1000 | 500 - 1000 |

* Range fo *D* dependent on parameter values of lower order models.

**Gaussian probability distributions for contributions of the three compartments of the ESL microstructure model.**

Values for mean and sigma of the Gaussian distributions for the ESL model were based on literature data where available.

- For epithelium *D* mean we used 0.3 µm^2^/ms, which is the ADC reported for Gleason grade 5+5 cancer (Manetta et al. 2019) characterised by very high-density epithelial cells with minimal stroma and lumen space.
- For epithelium *T2* mean we used 60 ms, based on the short *T2* component reporrted from lumen water imaging (Sabouri et al. 2017), although we note that this component is not specifically assigned to intracellular water.
- For lumen *D* mean we used 2.5 µm^2^/ms, which is the ADC reported for brain ventricles (Sener 2001, Ellingson et al. 2015).
- For lumen *T2* we used 550 (mean) ± 200 ms (sigma), based on the long *T2* component reporrted from lumen water imaging (Sabouri et al. 2017).
- For stroma *D* we initially used D = 0.8 ± 0.5 µm^2^/ms based on brain white matter (Ellingson et al. 2015), and *T2* = 80 ±50 ms. However, these values produced (in all patient data sets tested) ESL predictions with implausibly high stroma fractional volumes that were inconsistent with post-surgery histology.

| 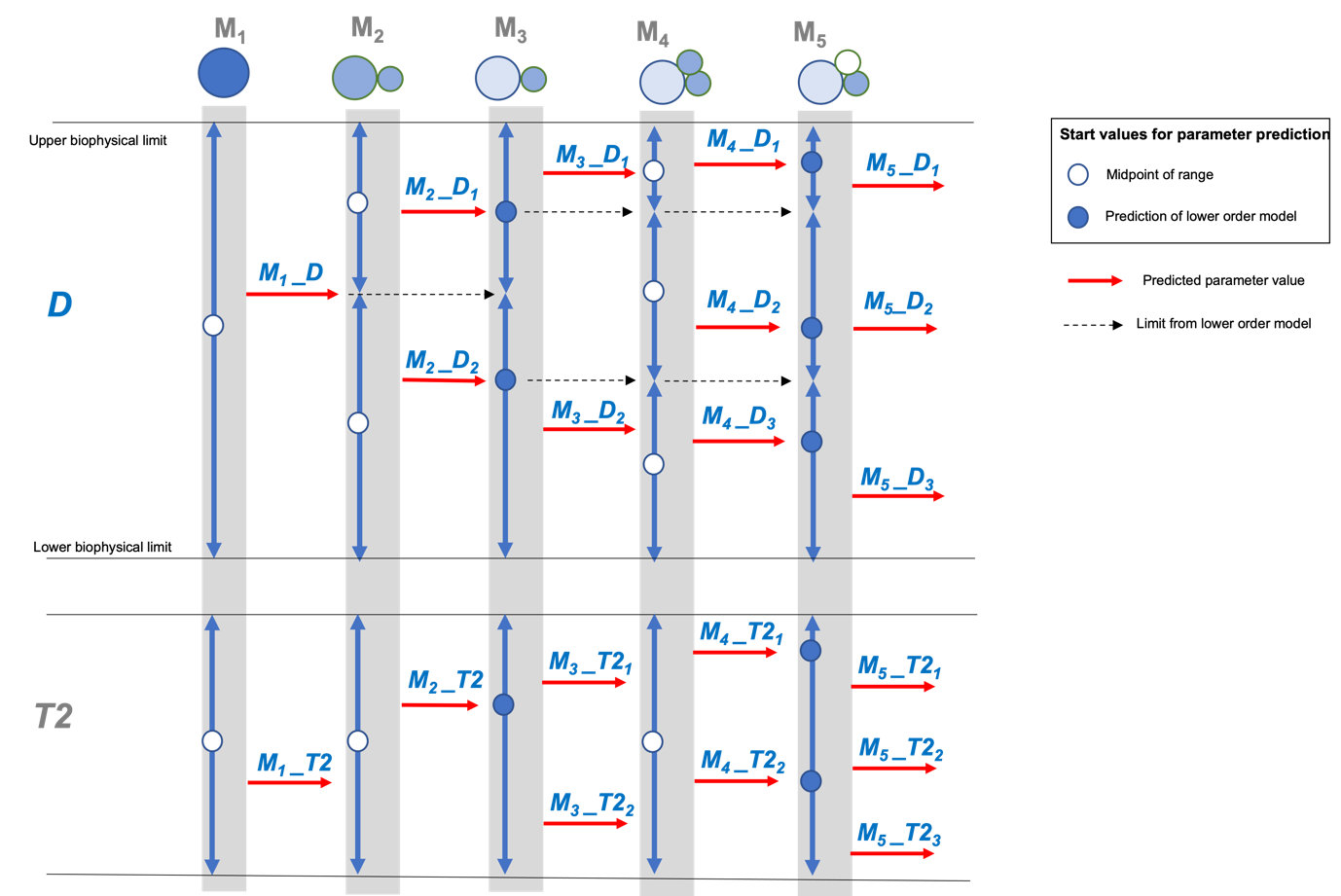 |
| --- |
| **Supplementary Fig. S2. Phenomenological model fitting sequence**  Five nested models with complexity (number of water pools and/or fitted parameters) increasing from left to right. The D and T2 ranges along models and pools are indicated by the blue arrows. For certain models, the starting point during fitting optimisation was chosen as the midpoint of a given range (white circles) or, in some others, by the estimated parameters (red arrows) of a lower order model (blue circles). Only the D ranges were limited across pools (dashed black arrows), while the full T2 range was always allowed across models and pools. |

**Results**

**ADC, T2 and volume fraction maps from patients 1-3 in main text (Figs. 2-4)**

To provide further detail on the D and T2 voxel values for patients 1, 2 and 3, in main text (Figs. 2- 4), the non-volume weighted maps are shown in Supplementary Fig. S3 top, middle, and bottom, respectively. Here, the ADC, T2 and volume fraction maps are plotted independently. It should be noted that for Model 1, no volume fraction map is shown as, for this model, only one compartment is assumed and, therefore, all volumes are equal to 1.

| 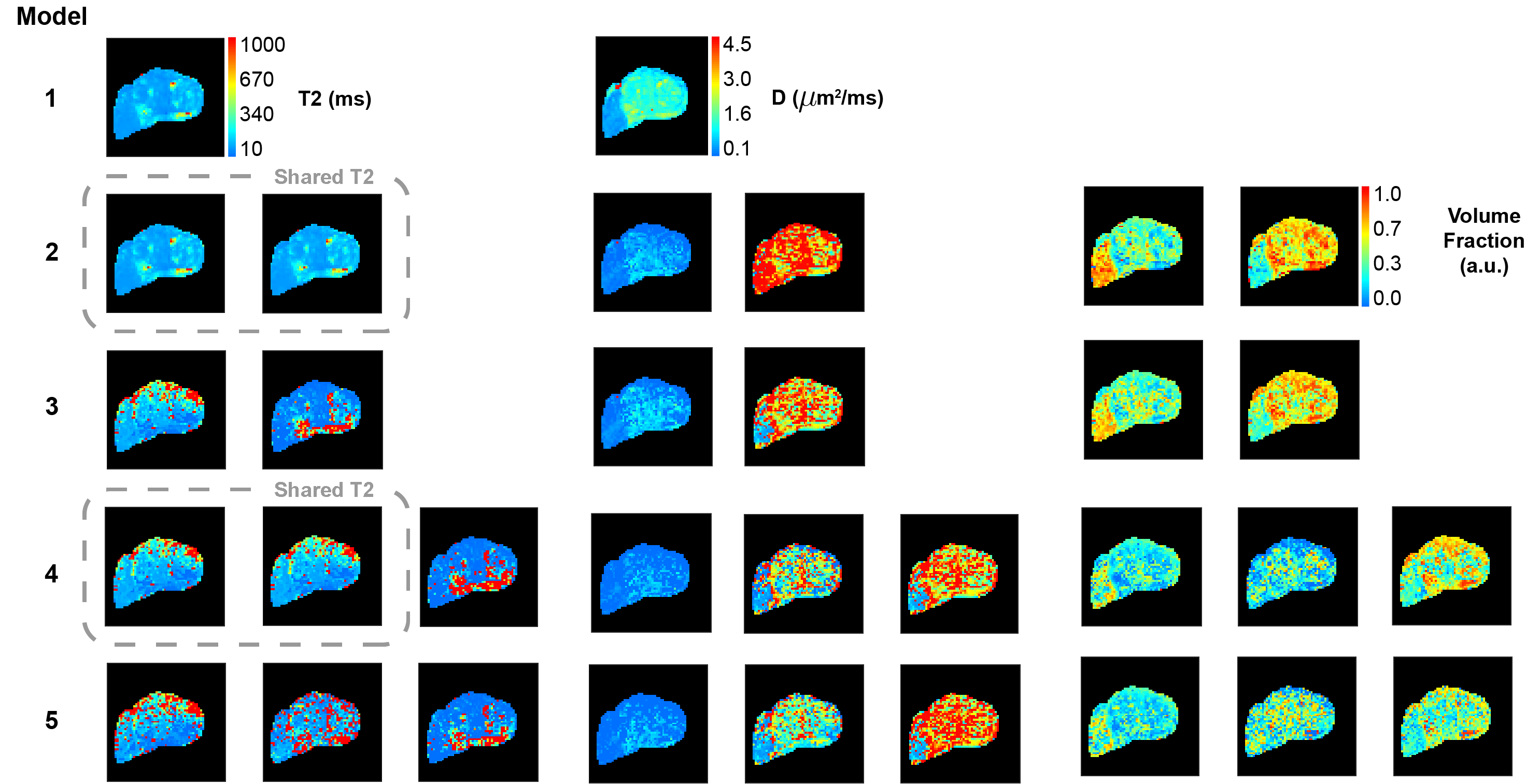 |
| --- |
| **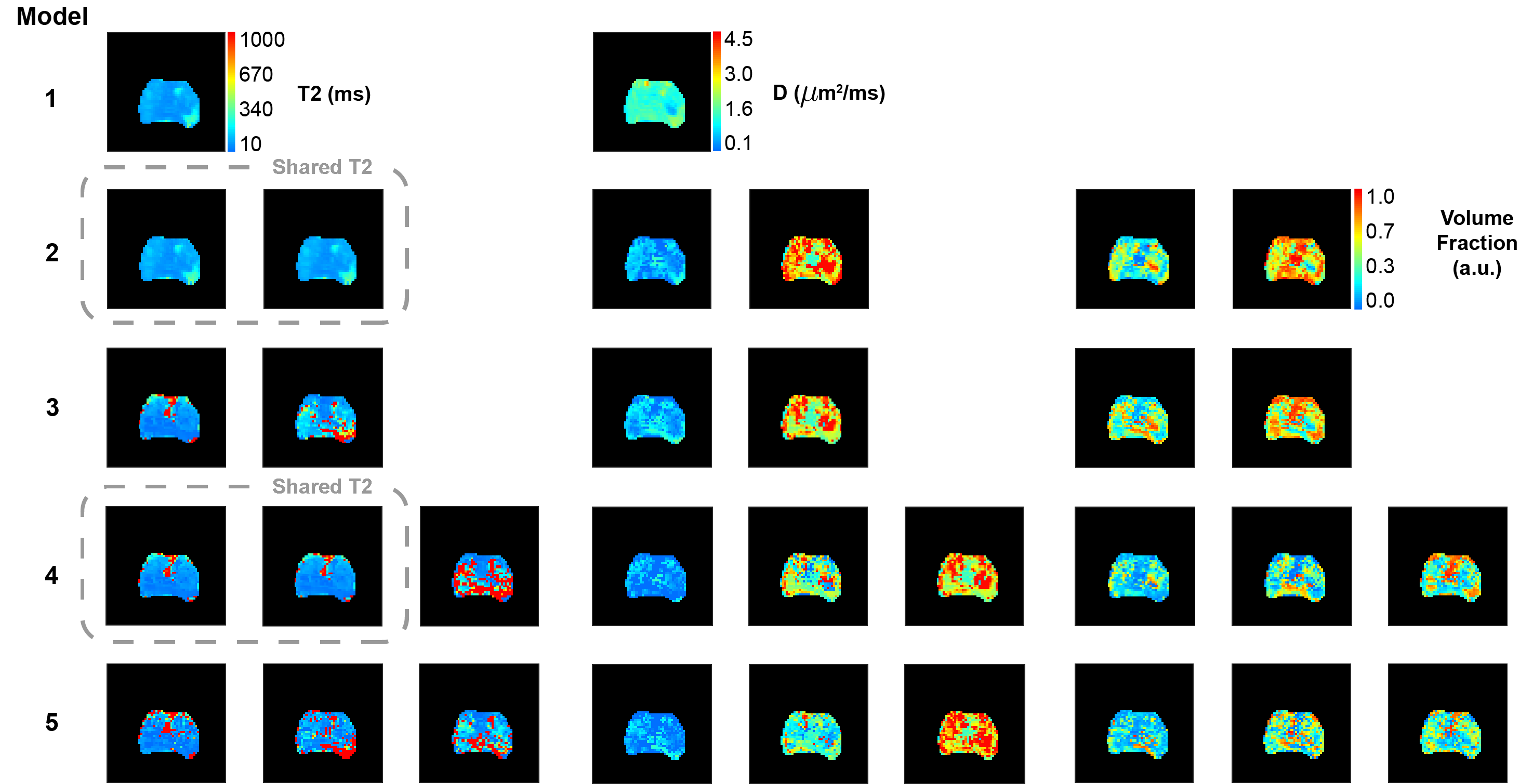** |
| **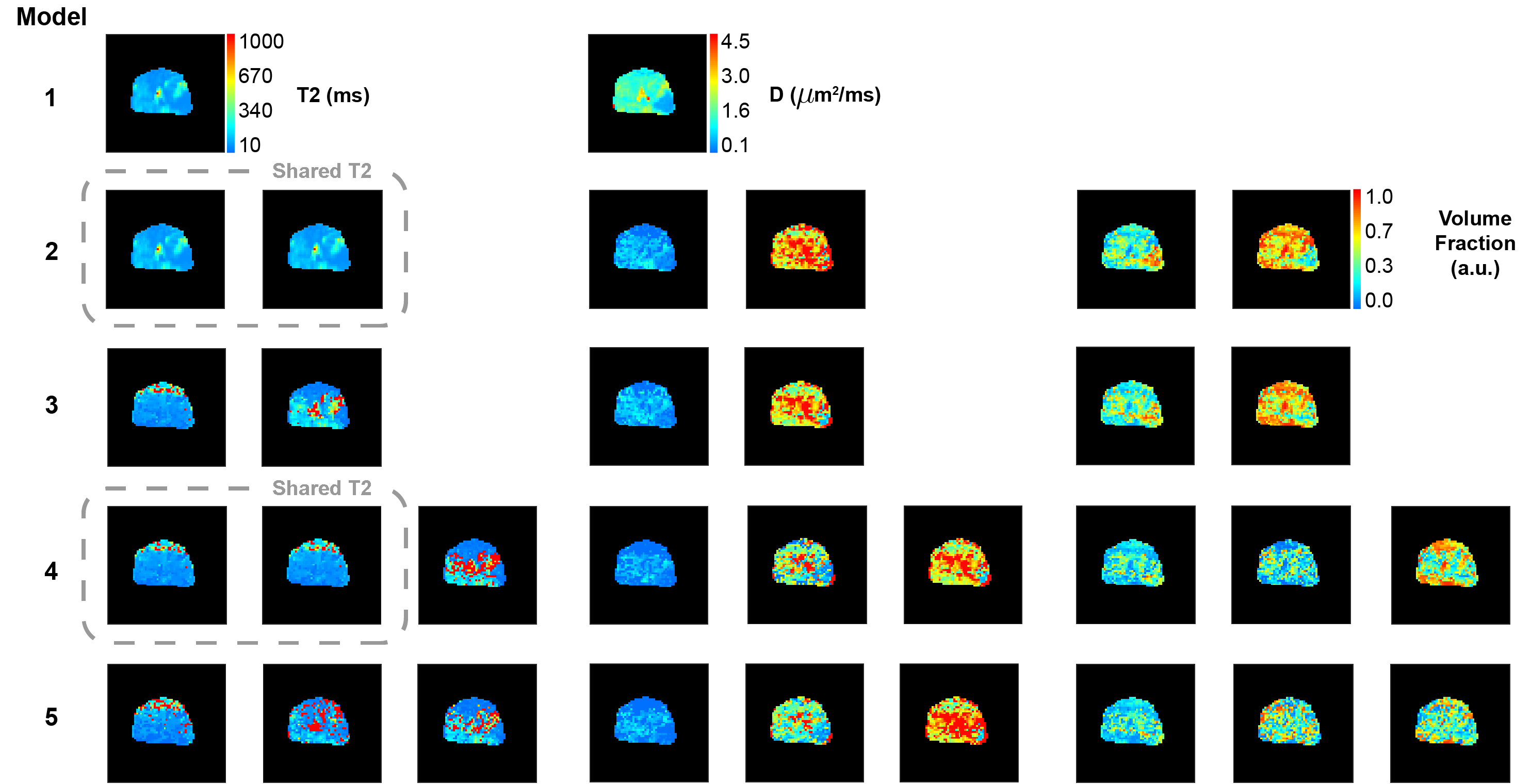** |
| **Supplementary Fig. S3. ADC, T2 and fractional volume maps for patients in main text: Patient 1 (top; yellow box), patient 2 (middle; green box) and patient 3 (bottom; orange box).** Water pools sharing the same T2 value are enclosed in dashed grey boxes. |

**References**

Chatterjee, A. et al. Validation of prostate tissue composition by using hybrid multidimensional MRI: correlation with histologic findings. Radiology 302, 368 (2022).

Ellingson, et al. "Diffusion MRI quality control and functional diffusion map results in ACRIN 6677/RTOG 0625: a multicenter, randomized, phase II trial of bevacizumab and chemotherapy in recurrent glioblastoma." *International journal of oncology* 46.5 (2015): 1883-1892.

Gudbjartsson, H. & Patz, S. The rician distribution of noisy mri data. Magn. resonance medicine 34, 910–914 (1995)

Manetta, R, et al. "Correlation between ADC values and Gleason score in evaluation of prostate cancer: multicentre experience and review of the literature." Gland surgery 8. Suppl 3 (2019): S216.

Sener, R. N. "Diffusion MRI: apparent diffusion coefficient (ADC) values in the normal brain and a classification of brain disorders based on ADC values." *Computerized medical imaging and graphics* 25.4 (2001): 299-326.

Sabouri, S. et al. MR measurement of luminal water in prostate gland: Quantitative correlation between MRI and histology. J. Magn. Reson. Imaging 46, 861–869 (2017).
